# Pregnancy-Induced Amelioration of Muscular Dystrophy Phenotype in *mdx* Mice via Muscle Membrane Stabilization Effect of Glucocorticoid

**DOI:** 10.1371/journal.pone.0120325

**Published:** 2015-03-16

**Authors:** Yuko Shimizu-Motohashi, Yoko Asakura, Norio Motohashi, Nandkishore R. Belur, Michael G. Baumrucker, Atsushi Asakura

**Affiliations:** Stem Cell Institute, Paul and Sheila Wellstone Muscular Dystrophy Center, Department of Neurology, University of Minnesota Medical School, Minneapolis, MN, United States of America; Medical College of Georgia, UNITED STATES

## Abstract

Duchenne muscular dystrophy (DMD), the most common and severe type of dystrophinopathy, is an X-linked recessive genetic disease caused by the absence of dystrophin, which leads to fragility and vulnerability of the sarcolemma to mechanical stretching with increased membrane permeability. Currently, glucocorticoids such as prednisolone are the only medication available for DMD. However, molecular pathways responsible for this effect are still unclear. In addition, it remains unclear whether sex-related factors, including pregnancy and the postpartum period, affect the phenotype of dystrophinopathy. Here, we report the amelioration of muscle membrane permeability in the diaphragm muscle of pregnant and postpartum, but not in nulliparous, *mdx* mice, an animal model for DMD, during the physiological surge of corticosterone, the most abundant glucocorticoid in rodents. Cultures of single muscle fibers and myotubes isolated from *mdx* mouse diaphragm demonstrate resistance to hypo-osmotic shock when treated with corticosterone but not with estradiol or progesterone. This corticosterone-mediated resistance was diminished by an antagonist of corticosterone, indicating that the glucocorticoid-glucocorticoid receptor axis plays a role in this membrane stabilization effect on muscle. Moreover, subcutaneous injection of corticosterone into *mdx* mice showed decreased membrane permeability. This is the first report to demonstrate that pregnancy-related resistance to muscle fiber damage in *mdx* mice due to the membrane stabilization effect of corticosterone. We also propose that this membrane stabilization effect is exerted through annexin A1 up-regulation as the molecular mechanisms of glucocorticoid effects on DMD muscle. Furthermore, single muscle fiber culture studies provide a sensitive chemical screening platform for muscular dystrophies.

## Introduction

Sex differences in disease susceptibility and severity have been reported in several neuromuscular and neurological disorders and are caused by a combination of hormonal, genetic, and epigenetic factors. For example, sex-related differences in multiple sclerosis (MS) and Amyotrophic lateral sclerosis (ALS) result from hormonal modulation during puberty and pregnancy [1], [2]. Duchenne muscular dystrophy (DMD), the most common and severe type of dystrophinopathy, is an X-linked muscular disorder affecting approximately 1 in 3600–6000 live male births which manifests progressive muscle weakness with early mortality due to cardiac and respiratory failure [3]. DMD is caused by a mutation in the *dystrophin* gene located on Xp21 which leads to the absence or decreased level of the dystrophin protein [4], [5]. Dystrophin protein links the intra-cellular cytoskeleton to the dystrophin-associated protein complex (DAPC) [6]. In the absence of dystrophin, the sarcolemma is more fragile and muscles are vulnerable to mechanical stretching [7]. However, it remains unknown whether sex-related factors including pregnancy and the postpartum period affect the phenotype of dystrophinopathy. Symptomatic female DMD carriers display 10–20 times higher than normal creatine kinase (CK) levels and dystrophin-associated dilated cardiomyopathy [8]. Recent reports have revealed a mosaic pattern of dystrophin expression in female DMD carriers with varying degrees of DMD symptoms that may have been previously dismissed [9]. In addition, the stressful process of pregnancy and parturition remains uncomplicated in at least some of the female DMD carriers.

Several therapeutic approaches such as induction of dystrophin, muscle replacement, vascular flow regulation, and fibrosis inhibition have been investigated for the treatment of DMD [10]. Although some successful results in animal models and trials in humans have been reported [3], to date, there is no established curative therapy for DMD. Currently, glucocorticoids such as prednisolone are the only medication available that slows the decline in muscle strength and function in DMD [3]. Although the clinical efficacy of glucocorticoids is established, it is still unclear how they act and which molecular pathways provide efficacy in DMD [11]. It has been reported that glucocorticoids reduce muscle degeneration [12], cell death [13], [14], proteolysis [15], and muscle damage [16] while others have reported positive effects on myogenic differentiation [17], [18]. Glucocorticoids are also potent immune modulators and have been shown to be anti-inflammatory [19], [20], immunosuppressive [21], reduce intracellular Ca^2+^ influx [18], [22], [23], attenuate fibrotic response [24], modulate myofiber type [25], and stabilize muscle membrane [18], [22], [23], [26]. More recent studies demonstrated calcineurin/NFAT pathway activation [27], inhibition of NF-κB signaling [20], and increased integrin α7 and laminin α2 [26] as the molecular pathway of the effect.

Herein, we tested whether pregnancy and the postpartum period affect the dystrophinopathic phenotype of *mdx* mice, an animal model of DMD.

## Materials and Methods

### Animals

DMD model *mdx* ^*5cv*^ (B6Ros.Cg-Dmd*mdx*-5Cv/J) mice and wild-type C57BL/6J mice were obtained from the Jackson Laboratory. Genotyping to detect the mutated *mdx* ^*5cv*^ allele was performed by PCR using the primers shown in S1 Fig. The PCR product DNA was digested with *Dra*III restriction enzyme (New England Biolabs) [28]. For the pregnant *mdx* mice, identification of the vaginal plug was counted as gestation day (GD) 0.5. The pups were separated from their mother on postpartum day (PD) 4. The animals were housed in an SPF environment and were monitored by the Research Animal Resources (RAR) staff of the University of Minnesota until they reached experiment age. Animal numbers were based on previous work in our lab [28]. The animals were provided access to drinking water and standard chow ad libitum and monitored daily prior to the experiments. All protocols were approved by the Institutional Animal Care and Usage Committee (IACUC) of the University of Minnesota. The animals were euthanized by appropriate means (CO2/O2 inhalation or KCl injection after being anesthetized with IP injection of pentobarbital. These methods are consistent with the recommendations of the Panel of Euthanasia of the American Veterinary Medical Association.

### Histological analysis

Evans blue dye (EBD) (1%, Sigma-Aldrich) was intraperitoneally injected [29]. Twenty hours after injection, muscle tissues were harvested. Microscopic images were captured by a DP-70 digital microscope camera attached to BX51 microscope with 4 x and 10 x UPlanFLN objectives (all from Olympus). Photoshop CS2 (Adobe Systems) was used for image processing. Freshly dissected tissues were frozen and sectioned at 8 μm thickness by cryostat (Leica CM1900). Sections were stained with hematoxylin and eosin (HE) for quantification of fibers with centrally located nuclei (CLN) and fiber diameters. Sections were also stained with Alizarin red (Sigma-Aldrich) and Sirius red (Sigma-Aldrich) for the assessment of calcification and fibrosis, respectively. The quantifications were done using the entire section area. Quantitative analysis was performed by the ImageJ software from the NIH.

### Immunostaining

Muscle sections were labeled with anti-CD31 (eBioscience), followed by biotin-conjugated anti-rat mouse IgG secondary antibody with Vectastain Elite ABC Kit (Vector Laboratories), and then stained with 3-amino-9- ethylcarbazole (AEC) (Sigma-Aldrich). Muscle sections were also labeled with anti-CD45 (eBioscience), anti-slow myosin heavy chain (MHC) antibody (Sigma-Aldrich) followed by Alexa 488-conjugated anti-rat IgG. For dystrophin immunostaining, M.O.M kit (Vector Laboratories) was used for blocking prior to anti-dystrophin antibody (NCL-DYS2)(Leica Biosystems) labeling, followed by Alexa 488-conjugated anti mouse IgG. Anti-annexin A1 antibody (Life Technologies) and anti-sarcomeric myosin heavy chain (MF20, Developmental Study for Hybridoma Bank), followed by Alexa 488-conjugated anti-mouse IgG and Alexa 568-conjugated anti-rabbit IgG secondary antibodies (Molecular Probes), were used for myotube cultures. Sections and cell cultures were also nuclear-stained with DAPI (4’,6-diamidino-2-phenylindole) (Sigma-Aldrich) and mounted with Dako fluorescence mounting media (Dako). Microscopic images were captured by a DP-70 digital camera attached to BX51 microscope with 20 x and 40 x UPlanFLN objectives (all from Olympus). Photoshop CS2 (Adobe Systems) was used for image processing. For quantification of CD45-positive area, slow MHC-positive fibers and dystrophin-positive revertant fibers, 14 randomly selected fields were analyzed. The counting of CD31-positive cells was done for the entire section area. Quantitative analysis was performed by the ImageJ software from the NIH.

### Western blotting

Muscle proteins were extracted using lysis buffer containing 70 mM Tris-HCl (pH 6.8), 10% sodium dodecyl sulfate (SDS), 5 mM EDTA, and 5% 2-mercaptoethanol. The protein concentration of the fractions was determined by Micro BCA Protein Assay Reagent Kit (Thermo Scientific). Fifty μg of protein was loaded onto 6% SDS polyacrylamide gel for electrophoresis. Utrophin and loading control β-tubulin were detected by western blotting with anti-utrophin antibody (Developmental Study for Hybridoma Bank) and anti-β-tubulin antibody (Sigma-Aldrich) followed by anti-mouse IgG-HRP (Bio-Rad). Quantitative analysis was performed by the ImageJ software from the NIH.

### Single muscle fiber isolation from diaphragm and hypo-osmotic shock

Diaphragm of 2-month-old mice was carefully dissected by cutting along the rib line, sternum, and spine. The dissected tissue was incubated in 0.2% collagenase type I (Sigma-Aldrich) in Dulbecco’s Modified Eagle Medium (DMEM) at 37°C for 2–2.5 hours. Digested diaphragm was then transferred to a plate containing only DMEM and was gently swirled to release the muscle fibers as described previously [30], [31]. For hypo-osmotic shock experiment, single muscle fibers were picked up and transferred with pipette to 48 well plate, 4 fibers per well, with DMEM supplemented with 10% fetal bovine serum (FBS). Corticosterone (28.8 μM), 17β-estradiol (27.5 μM), progesterone (27.6 μM), and mifepristone (58 μM) (all from Sigma-Aldrich) were added to fiber cultures, which were maintained at 37°C in a 5% CO_2_ incubator until the hypo-osmotic shock procedure. For hypo-osmotic shock medium, 10% FBS DMEM was diluted with water as described previously [32]. Hypo-osmotic shock medium was added to produce 100%, 75%, 50%, or 25% (medium/total volume). After the incubation at 37°C and 5% CO_2_ for 30 minutes, the number of live fibers was counted under stereo microscope (Olympus SZ61). The fibers which were long, shiny, and lucent were counted as live fibers, whereas dark, hyper-contracted fibers were counted as dead as described previously [31].

### Myotube hypo-osmotic shock

Satellite cell-derived primary myoblasts were isolated from adult female diaphragm of 2-month-old wild-type and *mdx* mice as described previously [33]. Cells were maintained on collagen-coated dishes in myoblast growth medium consisting of HAM’s F-10 medium supplemented with 20% FBS, 10 ng/ml basic FGF (Life Technologies) and 1% penicillin/streptomycin (Life Technologies). Four days after differentiation conditions (DMEM supplemented with 5% horse serum and 1% penicillin/streptomycin) which induced myotube formation, Corticosterone (28.8 μM) alone, or with mifepristone (58 μM), was added 20 hours before hypo-osmotic shock experiments. For hypo-osmotic shock medium, 10% FBS DMEM diluted with water (1:1) was used. After the incubation at 37°C and 5% CO_2_ with hypo-osmotic shock medium for 1 hour, damaged myotubes were quantified by the number of Trypan blue (Life Technologies)-positive cells as described previously [34].

### Viral vector preparation and infection

The pMX-based retroviral vector (Cell Biolabs) for annexin A1 expression was constructed by insertion of a mouse *annexin A1* cDNA fragment amplified from myotube cDNA by AccuPrime Pfx *SuperMix (Life technolies)* into the EcoRI site of the pMX vectors. The empty pMX and pMX-annexin A1 retroviral vectors were transfected into PlatE cells (Cell Biolabs) using TransIT 2020 Reagents (Mirus Bio). The retrovirus supernatants, lentiviral shRNA for *annexin A1* and control lentiviral shRNA (both from Santa Cruz Biotechnologies) were used for viral infection to primary myoblasts seeded at a density of 2 x 10^5^ cells per 3 cm plates with 10 μg/ml of polybrene (Millipore) as described previously [35].

### Corticosterone, 17β-estradiol, and progesterone injection into *mdx* mice

Corticosterone (12.5 mg/kg/day), 17β-estradiol (20 μg/kg/day), progesterone (40 μg/kg/day) (all from Sigma-Aldrich) were dissolved in ethanol:corn oil (1:9), and 100 μl was subcutaneously injected once per day in the morning for 2 days. The tissues were harvested one day after the final injection. EBD was intraperitoneally injected 20 hours prior to harvesting the tissue.

### Quantitative real-time PCR

Total RNA was extracted from diaphragm muscle and isolated diaphragm muscle myotube cultures using Trizol reagent (Life technologies). First strand cDNA was generated using Transcriptor First Strand cDNA Synthesis kit (Roche Applied Science), and GoTaq qPCR Master Mix (Promega) was used for qPCR with the specific primers (S1 Fig.). The expression levels of genes were quantified on a Mastercycler ep realplex real-time PCR machine (Eppendorf). Each sample was run in triplicates. The mRNA expression was normalized against the expression of β-actin, and GAPDH for myotubes and diaphragm muscle, respectively, and the mRNA level was quantified by comparative C*t* (ΔΔCt) method [36].

### Statistics

All data are presented as mean±SEM. At least three independent experiments were performed. Comparison between groups was done by Student’s t-test using a two-tailed distribution. Data were considered statistically significant at *p<0.05 and **p<0.01.

## Results

### Diaphragms of *mdx* mice show amelioration of muscle membrane permeability during pregnancy and the postpartum period

**Fig 1:**
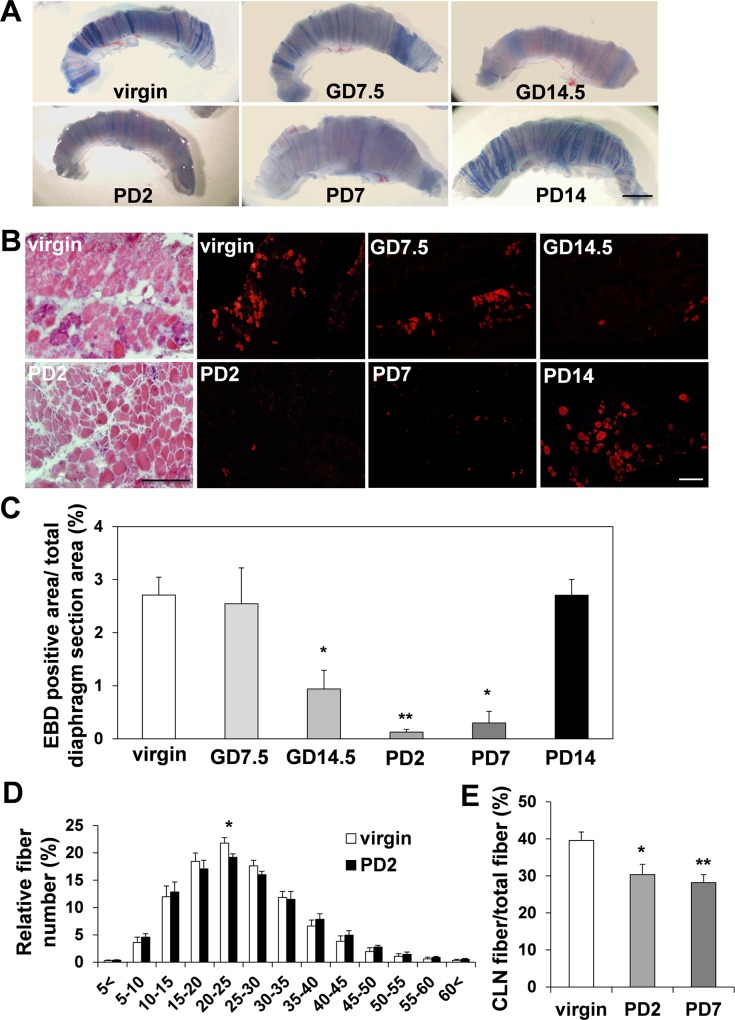
Muscle fiber characterizations in the diaphragm from virgin, pregnant and postpartum *mdx* mice. (A) Diaphragm from 2.5-month-old virgin and pregnant female *mdx* mice with EBD injection. Gestation day 7.5 (GD7.5) and GD14.5 mean 7 and 14 days after the recognition of vaginal plug in mated female mice, respectively. Postpartum day 2 (PD2), PD7 and PD14 mean 2, 7 and 14 days after parturition, respectively. Bar (2 mm) (B) Diaphragm sections examined by fluorescence microscopy. Bars (200 μm). (C) The EBD uptake at GD14.5 (n = 3), PD2 (n = 4), PD7 (n = 3) and the virgin female *mdx* mice (n = 5). (D) Fiber size (μm) distribution in virgin (n = 4) and PD2 *mdx* mice (n = 4). (E) Histogram of number of fibers with centrally located nuclei (CLN) in virgin (n = 9), PD2 (n = 7) and PD7 (n = 5) *mdx* mice.

We noticed that pregnant and postpartum 2.5-month-old female *mdx* mice at gestation day (GD) 14.5 (0.94±0.35%), postpartum day (PD) 2 (0.12±0.05%), and PD7 (0.29±0.22%) have decreased Evans blue dye (EBD) uptake in their diaphragms compared to virgin (2.71±0.34%) and GD7.5 (2.55±0.68%) *mdx* mice (Fig. 1A, 1B, 1C). EBD is a dye widely used to study cellular membrane permeability, and dystrophic muscle fibers.

The dye binds to serum albumin and leaks into muscle fibers that are damaged by rupture of the plasma membrane [37]. Therefore, we concluded that pregnancy and the postpartum period are affecting muscle membrane permeability in the diaphragm of *mdx* mice. Although the area of fibrosis analyzed after Sirius red staining showed comparable results between the virgin and the PD2 *mdx* mice (S2A Fig.), fiber size distribution analyzed after HE staining (Fig. 1D) showed slightly increased fiber diameters in PD2 *mdx* mice compared to virgin *mdx* mice, indicating increased muscle fiber stability in pregnant/postpartum *mdx* mice. We also counted the number of centrally located nuclei (CLN), which was expected to be decreased in pregnant/postpartum *mdx* mice due to increased muscle membrane stability and decreased muscle turnover. There was a trend of having fewer CLN at PD2 (30.33±2.78%) and significantly lower at PD7 (298.18±2.17%) compared to virgin mice (39.58±2.28%) (Fig. 1E).

It has been reported that depletion of inflammatory T cells from *mdx* mice could significantly reduce the muscle pathology [38]. However, there was no significant difference in the area with CD45-positive inflammatory cell invasion between diaphragm of PD2 and virgin *mdx* mice, suggestive of muscle cell permeability amelioration not being due to the decreased inflammatory cell numbers (S2B Fig.). In DMD patients, fast muscle fibers are preferentially affected [39], and reduced levels of contractile damage is induced by a shift in fiber type toward slow-twitch [32]. However, immunostaining showed no significant change in the number of slow myosin heavy chain (MHC)-positive fibers between of PD2 and virgin *mdx* mice diaphragms (S2C Fig.). We have recently demonstrated that increased vascular density can decrease the amount of EBD-positive fibers and ameliorate the dystrophic phenotype in *mdx* mice [28]. The quantification of CD31-positive blood vessel density did not reveal a significant difference between the virgin and the PD2 *mdx* mice (S2D Fig.). The dystrophin-related protein utrophin can functionally compensate for the lack of dystrophin in the *mdx* mice [40]. However, no significant change in utrophin was observed between the diaphragm of PD2 and virgin *mdx* mice PD2 mice by the western blotting analysis (S2E Fig.). In addition, there was no increase in dystrophin-positive revertant fibers in PD2 *mdx* mice diaphragm (S2F Fig.). Since there was no significant change in inflammatory area, number of slow muscle fibers, vascular density, and expression of dystrophin and utrophin between the virgin and PD2 *mdx* mice, we concluded that the causative molecule which decreased EBD uptake in pregnant/postpartum *mdx* mice may have a stabilizing effect on the muscle membrane.

### Corticosterone-treated single muscle fibers isolated from diaphragm showed increased survival after hypo-osmotic shock

The concentration of corticosterone, estrogen and progesterone increase during pregnancy [41]. Studies have demonstrated that corticosterone is the most abundant glucocorticoid in rodents [41]. Also, studies on steroid hormones in rodents have shown that progesterone increases from early pregnancy, peaks around GD16, and declines to baseline by GD21, whereas estrogen also increases from early pregnancy but peaks around GD18-21 and declines rapidly thereafter [41], [42]. The concentration of corticosterone starts to rise around GD12, peaking at GD16-17, and remains elevated for a while after parturition [41], [43], [44]. In our pregnant/postpartum *mdx* mice, decreased EBD uptake was observed between GD14.5 and PD7, which matched the period of physiological elevation of serum corticosterone during pregnancy in rodents [41], [43], [44]. Therefore, we hypothesized that corticosterone may have a membrane stabilization effect in *mdx* mice diaphragm.

**Fig 2:**
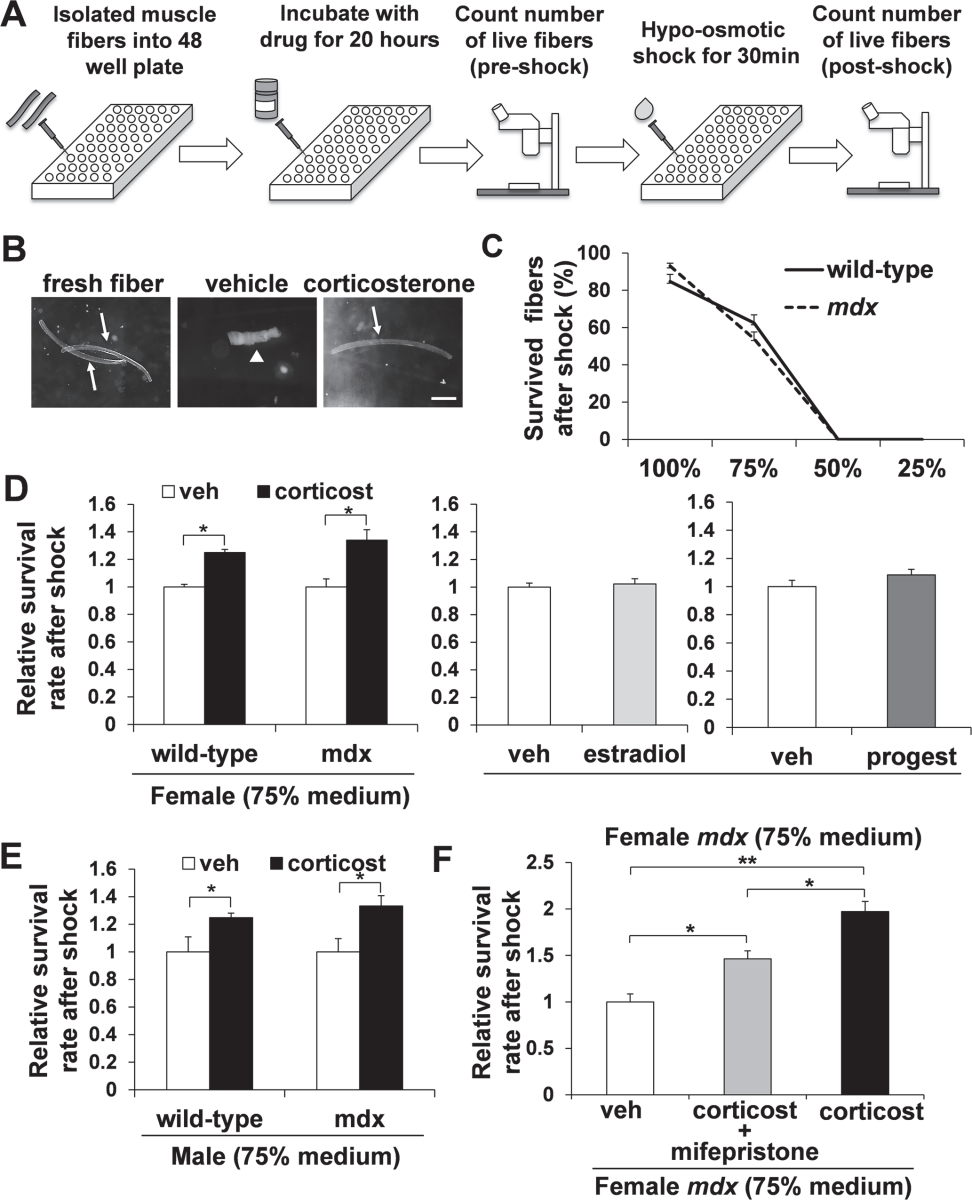
Screening of molecules with hypo-osmotic shock in single muscle fibers. (A) Schematic figure of hypo-osmotic shock. Single muscle fibers were incubated for 20 hours with vehicle, corticosterone, estradiol, or progesterone. The number of live fibers was compared prior to the hypo-osmotic shock and after 30 min of incubation with hypo-osmotic shock medium. (B) Representative images of fibers before and after hypo-osmotic shock with 75% medium following treatment with or without corticosterone. Live and dead fibers are indicated with arrows and arrow head, respectively. Bar (250 μm). (C) Survival curves of wild-type (solid line) and *mdx* (broken line) female fibers 30 min after hypo-osmotic shock with 100%, 75%, 50% or 25% medium following with 20 hours preincubation (n = 5–6). (D) The relative survival rate of fibers from female wild-type (n = 11) and *mdx* (n = 9) mice treated with or without corticosterone after hypo-osmotic. The relative survival rate after hypo-osmotic shock in estradiol or progesterone treated fibers in *mdx* female mice (n = 5). (E) The relative survival rate of fibers from male wild-type (n = 5) and *mdx* (n = 5) mice treated with or without corticosterone. (F) The fibers from female *mdx* mice treated with vehicle, corticosterone or corticosterone and mifepristone, followed by hypo-osmotic shock (n = 4).

It has been reported that hypo-osmotic shock can serve as a model for mechanical stress on the cell membrane-skeletal complex by creating the osmotic pressure exerted by the solutes inside the cell [45], [46]. We aimed to utilize the hypo-osmotic shock system to screen for the molecule that caused the muscle membrane stabilizing effect in pregnant and postpartum *mdx* mice (Fig. 2A, 2B).

Our data showed a clear decline in muscle fiber survival when the hypo-osmotic shock medium was used for both fibers isolated from diaphragm of 2-month-old female wild-type and *mdx* mice (Fig. 2C). There was no survival of fibers in 50% and 25% medium, but some survived fibers isolated from wild-type (62.5±4.5%) and *mdx* (54.0±3.6%) mice in 75% medium for 30 min (Fig. 2C). Therefore, we decided to apply 75% medium for the rest of the analysis. The muscle fibers were incubated with either vehicle, corticosterone (28.8 μM), estradiol (27.5 μM), or progesterone (27.6 μM) for 20 hours. Then, the fibers were subjected to either 100% medium as a control or 75% medium for 30 minutes, followed counting the number of survived fibers (S3 Fig.). The survival rate of the fibers after 20 hour incubation time was non-significant between the vehicle and the drug (corticosterone, estradiol or progesterone)-treated *mdx* fibers (S4A Fig.). Interestingly, *mdx* fibers incubated with corticosterone had a greater survival rate (1.34±0.08 fold increase) after hypo-osmotic shock than those treated with estradiol (1.02±0.04 fold increase) or progesterone (1.00±0.04 fold increase) (Fig. 2D) compared to vehicle alone. To examine the reproducibility of the data, and also to investigate whether this muscle protection effect is *mdx* specific, we isolated single muscle fibers from female wild-type mouse diaphragm. The number of live fibers after hypo-osmotic shock was significantly higher in corticosterone-treated fibers (1.25±0.06 fold increase) compared to vehicle alone (Fig. 2D). We also performed hypo-osmotic shock analysis after a shorter incubation period with corticosterone. Five hours of incubation with corticosterone did not result in increased survival rate after hypo-osmotic shock (S4B Fig.). Furthermore, the similar improved survival rate by corticosterone treatment after hypo-osmotic shock was also seen in muscle fibers isolated from male wild-type (1.25±0.03 fold increase) and *mdx* mice (1.33±0.07 fold increase) compared to vehicle alone (Fig. 2E). These results indicate that corticosterone may have a muscle membrane protective effect against the mechanical pressure and that corticosterone is the causative molecule that decreased the EBD uptake in pregnant/postpartum *mdx* mice diaphragm.

### The muscle membrane stabilization effect of corticosterone was diminished when treated with glucocorticoid receptor antagonist

Glucocorticoids can act through multiple mechanisms. In addition to binding to glucocorticoid receptors (GR), they can interact with the plasma membrane to exert physicochemical effects, which are important for resistance to and repair of membrane injury [11]. In order to elucidate which mechanism is responsible for the muscle membrane protective effect in corticosterone-treated fibers, we blocked GR with mifepristone, a GR antagonist [47] along with corticosterone treatment. The single muscle fibers were incubated with corticosterone and mifepristone (58 μM) for 20 hours and then subjected to hypo-osmotic shock. The fibers co-treated with corticosterone and mifepristone had a significantly lower survival rate (1.46±0.09 fold increase) after hypo-osmotic shock than the fibers treated with corticosterone alone (1.97±0.11 fold increase), compared to vehicle alone (Fig. 2F), suggesting the membrane stabilization effect of corticosterone through the GR binding process.

### Up-regulation of annexin A1 in corticosterone-treated muscle fibers and myotubes is able to protect against cell death after hypo-osmotic shock

**Fig 3:**
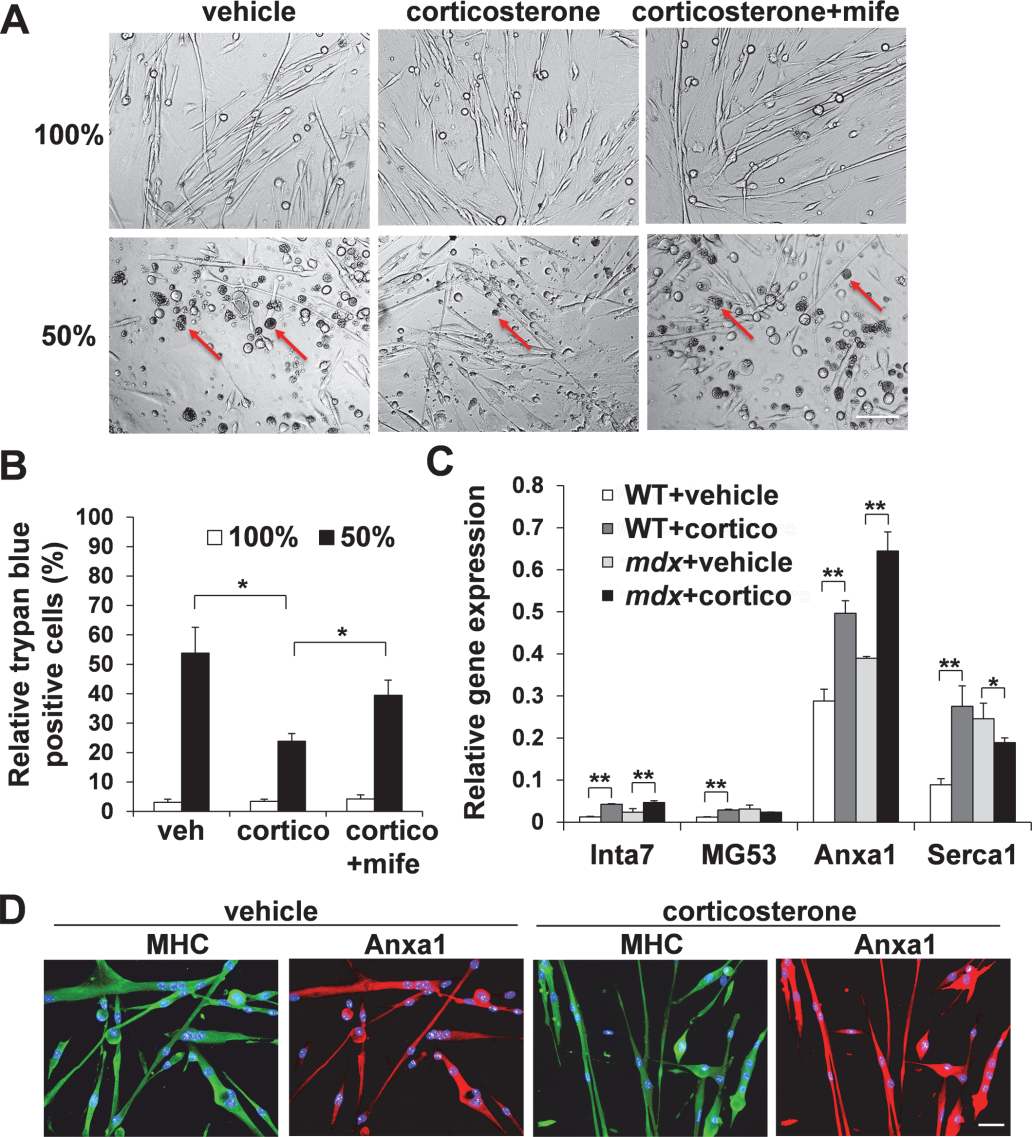
*Annexin A1* (*Anxa1*) is up-regulated after treatment with corticosterone. (A, B) Female *mdx* myotubes were incubated for 20 hours with vehicle, corticosterone or corticosterone and mifepristone. After 1 hour of incubation with 100% medium or 50% hypo-osmotic shock medium, relative dead cells were calculated as Trypan blue-positive cells (red arrows) per total cells (n = 3). Bar (200 μm). (C) mRNA levels of *Inta7*, *MG53*, *Anxa1*, and *Serca1* in vehicle or corticosterone-treated wild-type or *mdx* myotubes were quantified with real-time qPCR (n = 3). *β-Actin* was used for internal control. (D) Female *mdx* myotubes were incubated for 20 hours with vehicle or corticosterone, and stained for MHC (green) and annexin A1 (red). DAPI (blue) denotes all nuclei. Bar (50 μm).

Muscle fiber culture experiments showed the muscle membrane stabilization effect of corticosterone after hypo-osmotic shock. To confirm these results, we isolated primary myoblasts from diaphragm of 2-month-old female *mdx* mice and let them differentiate into myotubes under differentiation conditions. After 20 hours incubation with vehicle alone, corticosterone, or corticosterone and mifepristone, the myotubes were subjected to either 100% medium or 50% medium for 1 hour, followed by the counting of Trypan blue-positive damaged and necrotic cells as described previously [34]. Similar to the muscle fiber cultures, myotubes incubated with corticosterone had a significantly lower level of cell death (23.8±2.6%) compared to myotube cultures incubated with vehicle alone (53.8±8.8%) or corticosterone and mifepristone (39.4±5.2%) in 50% medium (Fig. 3A, 3B).

**Fig 4:**
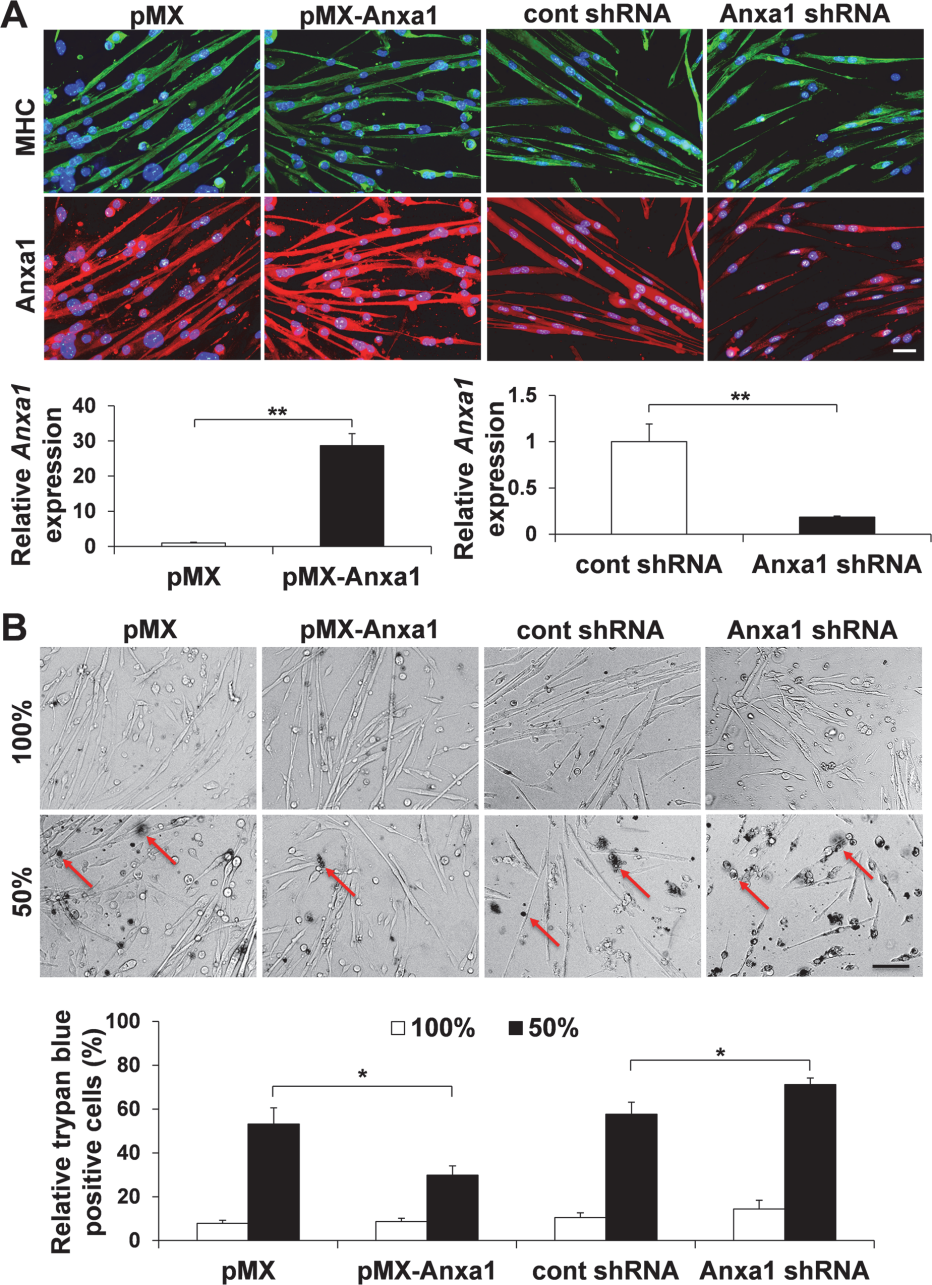
Over-expression of annexin A1 can protect muscle fiber death in hypo-osmotic shock. (A) Female *mdx* myoblasts were infected with empty retroviral vector (pMX), annexin A1 retroviral vector (pMX-Anxa1), control lentiviral shRNA vector or annexin A1 lentiviral shRNA vector. After myotube formation, cells were stained for MHC (green) and annexin A1 (red). DAPI (blue) denotes all nuclei. Bar (50 μm). Relative mRNA level of *annexin A1* in *mdx* myotubes was also quantified with real-time qPCR after viral infection (n = 3). *β-Actin* was used for internal control. (B) Myotubes were incubated with 100% medium or 50% hypo-osmotic shock medium for 1 hour, and stained with Trypan blue (red arrows). Relative dead cells were calculated as Trypan blue-positive cells per total cells (n = 3). Bar (200 μm).

We speculate that the glucocorticoid-mediated muscle membrane stabilizing effect is caused by either up-regulation of proteins that function positively for membrane integrity, ion channel stabilization, or through enhancement of the membrane repair system. Integrin α7 (Inta7), mitsugumin 53 (MG53), and sarco/endoplasmic reticulum Ca^2+^ ATPase 1 (Serca1) have been reported to have roles in muscle membrane integrity, muscle membrane repair, and regulation of intracellular calcium concentration, respectively [48], [49], [50]. In addition, amongst the known molecular pathways that are modulated by glucocorticoids, annexin A1 (Anxa1) has been reported to regulate membrane repair in non-muscle system [51], [52]. However, it remains unclear whether annexin A1 is able to stimulate muscle fiber membrane repair after treatment with corticosterone. We quantified mRNA expression levels of *Inta7*, *MG53*, *Serca1*, and *annexin A1* with real-time. Among the four genes, *annexin A1* showed the highest expression levels in both wild-type and *mdx* myotubes before treatment, and they were further up-regulated after treatment with corticosterone (Fig. 3C). Immunostaining also confirmed the up-regulation of annexin A1 in *mdx* myotubes after treatment with corticosterone (Fig. 3D). Retroviral vector-mediated overexpression of *annexin A1* clearly showed up-regulation of annexin A1 expression (Fig. 4A).

Female *mdx* myotubes showed a significantly lower level of cell death (29.9±4.2%) (Fig. 4B) in *mdx* myotubes infected with retroviral *annexin A1* expression vector after hypo-osmotic shock, compared to the control empty vector infection group (53.2±7.3%). By contrast, lentiviral vector-mediated knockdown of *annexin A1* showed down-regulation of annexin A1 expression in MHC-positive *mdx* myotubes (Fig. 4A) and a significantly higher level of cell death (71.1±3.0%) (Fig. 4B) in *mdx* myotubes after hypo-osmotic shock, compared to the control empty vector infection group (57.7±5.5%). These results suggested that the membrane stabilization effect mediated by corticosterone is exerted through annexin A1 up-regulation.

### Male *mdx* mice injected with corticosterone displayed decreased EBD uptake in the diaphragm

**Fig 5:**
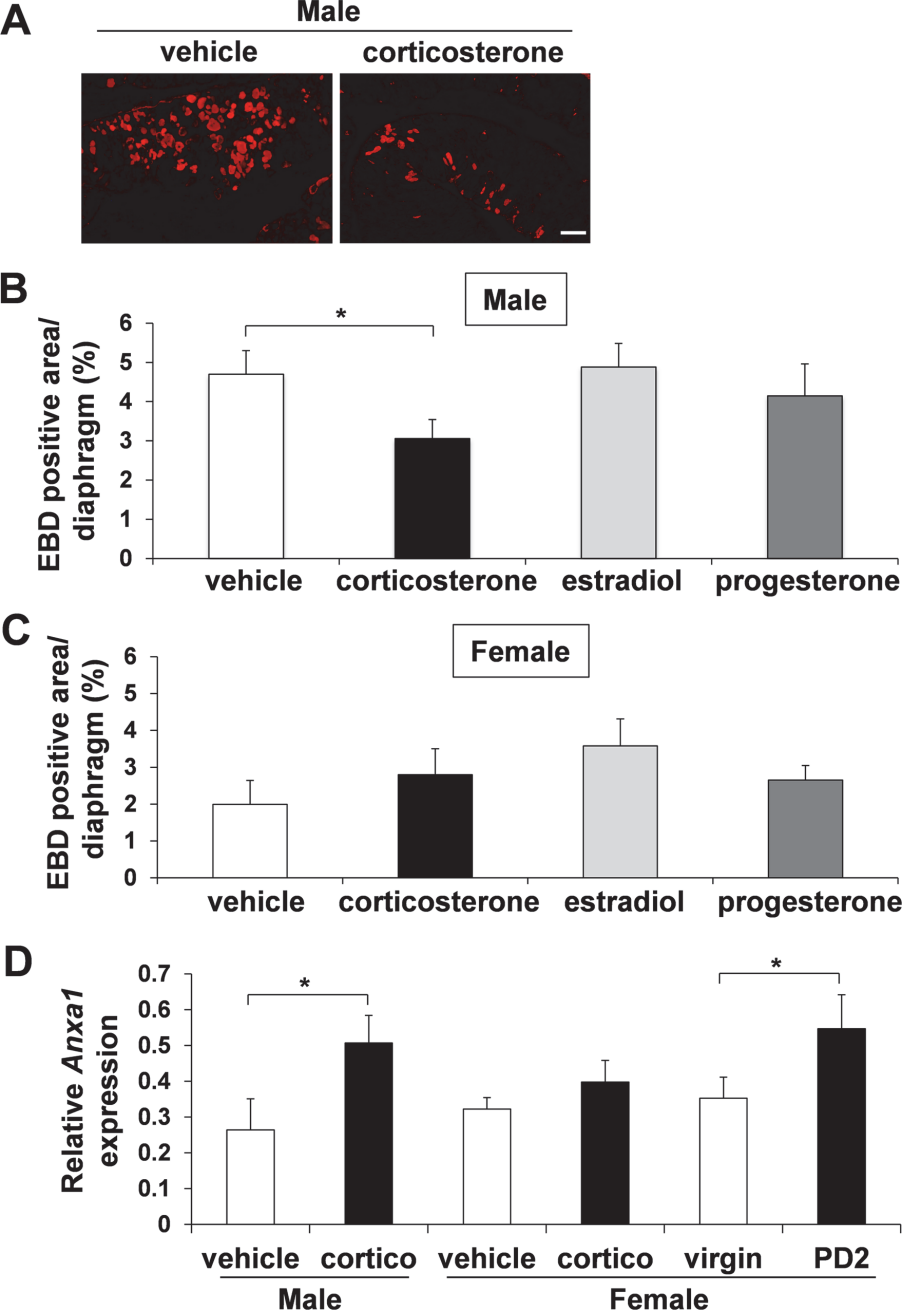
Decreased EBD uptake and increased annexin A1 up-regulation in the diaphragm of male *mdx* mice after injection with corticosterone or PD2 female *mdx* mice. EBD-positive area per total diaphragm section area was calculated after injecting male (A and B) and female (C) *mdx* mice with vehicle, corticosterone, estradiol, or progesterone for 2 days. Vehicle, n = 8 (male) and n = 4 (female); corticosterone, n = 9 (male) and n = 5 (female); estradiol, n = 9 (male) and n = 5 (female); progesterone, n = 9 (male) and n = 5 (female). Bar (200 μm). (D) mRNA levels of *Anxa1* in vehicle or corticosterone-treated mice, or virgin or PD2 female mice were quantified with real-time qPCR. Vehicle, n = 4 (male) and n = 6 (female); corticosterone, n = 6 (male) and n = 6 (female); n = 7 (virgin) and n = 6 (PD2). *GAPDH* was used for internal control.

To determine whether the findings from muscle fiber hypo-osmotic shock experiments can be recapitulated *in vivo*, corticosterone injection into *mdx* mice was performed to examine EBD uptake. Since *in vitro* assay demonstrated that the effect of corticosterone became visible after 20 hour incubation, we speculated that the corticosterone should show its efficacy within short term usage. We subcutaneously injected corticosterone to *mdx* mice for 2 days, and on the third day, diaphragms were resected to analyze EBD uptake. Male *mdx* mice injected with 12.5 mg/kg/day of corticosterone for 2 days showed significantly decreased EBD uptake in the diaphragm (3.06±0.49%) compared to vehicle alone (4.70±0.60%), 20 μg/kg/day estradiol (4.88±0.60%) and 40 μg/kg/day progesterone (4.15±0.82%) (Fig. 5A, 5B).

In female *mdx* mice, we could not see any significant change in EBD uptake between the corticosterone and the vehicle-treated group (Fig. 5C). Corticosterone injection in various doses and lengths (1.0 to 50 mg/kg for 2 days or 2 weeks) was also examined in female *mdx* mice, but there was no significant EBD uptake change in their diaphragms (data not shown). This may be partly due to the lower EBD uptake in the diaphragms of the control female *mdx* mice (1.99±0.65%) compared to those of the control male *mdx* mice (4.70±0.60%) (Fig. 5B, 5C). We also injected estradiol and progesterone at a dose that has been described previously [53] to female *mdx* mice, but again, no significant change in EBD uptake was observed between the estradiol, progesterone, and vehicle injected mice (data not shown). The decreased EBD uptake seen in male *mdx* diaphragm after injection with corticosterone was correlated with up-regulation of *annexin A1* transcription compared to vehicle injection group (Fig. 5D). The *annexin A1* up-regulation was also detected in diaphragm of PD2 female mice compared to virgin mice. However, female *mdx* diaphragm after injection with corticosterone did not significantly increase the expression level of *annexin A1*. Taken together, these results strongly indicate that corticosterone can display a muscle membrane stabilization effect *in vivo* possibly mediated by up-regulation of *annexin A1*, but the effect is significantly affected by the sex.

## Discussion

The first important finding in this paper is that muscle membrane permeability in the diaphragm was ameliorated in pregnant and postpartum, but not in virgin *mdx* mice during the period corresponding with the physiological surge of corticosterone. The second important finding is that corticosterone has a membrane stabilizing effect in *mdx* mouse diaphragm. Especially, single muscle fiber culture studies in combination with hypo-osmotic shock provide a sensitive chemical screening platform for muscular dystrophies. This corticosterone effect is likely exerted through the glucocorticoid-GR binding process. The third important finding is that annexin A1 is able to enhance muscle fiber membrane stability after treatment with corticosterone.

Since our hypothesis began from the observation of pregnant and postpartum mice, we analyzed the mice by sex. As a by-product of our research, we also noticed that the *in vivo* response to corticosterone in *mdx* mice muscle differs according to their sex. In this study, the injection of corticosterone could decrease the EBD uptake in male *mdx* mouse diaphragm but not in female mice. This may be partly due to the lower EBD uptake in the diaphragms of the control female *mdx* mice (1.99±0.65%) compared to those of the control male *mdx* mice (4.70±0.60%) (Fig. 5B, 5C). In addition, the sex difference in the response to corticosterone is well known in other organs such as the brain [54]. In studies that exposed rodents to stress which increased corticosterone levels in both sexes, suppressed hippocampal cell proliferation occurred in males but not in females [55], [56], while prolonged high corticosterone levels reduced cell proliferation in both sexes [55]. It also has been reported that receptor affinity of glucocorticoids in the female brain is half of that found in males, a difference that does not depend on circulating sex hormones [54]. In addition, females have higher baseline levels of corticosterone compared to males [55]. Since the *in vitro* experiment with isolated muscle fibers showed similar results of corticosterone having a membrane stabilizing effect in both sexes, the reason for the negative result in *in vivo* experiments for female *mdx* mice may be explained by the internal interference from the female-specific physiological events or suboptimal experimental design such as dose and treatment length in the current study.

There is a dynamic change in physiological status during pregnancy and the postpartum period. In this study, we focused on corticosterone as a contributor for muscle membrane stabilization during pregnancy and were able to show evidence to support the hypothesis. However, we acknowledge the possibility that other molecules and/or physiological changes caused the positive effect on diaphragm muscle membrane. Estrogen and progesterone have been suggested as a positive modulator for muscle physiology [57], and estrogen has been reported to have a protective effect in rat muscle against physiological damage. A recent study demonstrated that tamoxifen, a selective estrogen receptor modulator, could decrease plasma CK levels in *mdx* mice [58]. Also, estrogen is known to enhance the glucocorticoid response to acute stress in females [59], which implies that a synergetic effect of estrogen and corticosterone may have occurred in the muscle membranes of pregnant *mdx* mice. Not only hormonal changes, but also increased total volume of circulating blood during pregnancy [60] may have contributed to muscle membrane stabilization, as there is accumulating evidence that increased blood perfusion is beneficial to DMD muscle [28], [61], [62]. In addition, pregnancy may have beneficial effects from the fetus-derived secreted factors as well as fetal cells migrating into the maternal circulation as a fetal micro-chimerism [63]. Further research on pregnant and postpartum *mdx* mice may guide us to a novel discovery on DMD therapy.

There is no direct evidence showing the effects of glucocorticoids on muscle membrane stability in the past [11], although current studies have shown some relationship between glucocorticoids and muscle membrane stabilization. For example, prednisone could decrease the area of muscle damage after exercise in wild-type rats [16]. In addition, *mdx* myotubes have higher cytosolic Ca^2+^ concentration after hypo-osmotic shock compared to the wild-type, and that α-methylprednisolone could attenuate the rise of cytosolic Ca^2+^ [22]. In a study with *Caenorhabditis elegans* (*C*. *elegans*) carrying dystrophin-like gene (*dys-1*) mutation, prednisone could reduce the number of degenerating muscle cells [12]. Since *C*. *elegans* is not susceptible to inflammation, it was suggested that prednisone could have a direct effect on cell survival. Importantly, we demonstrate that a 20 hour incubation time, but not a shorter period of 5 hours, with corticosterone is required for achieving the muscle membrane stabilizing effect, consistent with the previous study, in which α-methylprednisolone could reduce the cytosolic Ca^2+^ influx into C2C12 cells, and the effect took at least 24 hours to become established [23]. On the other hand, the negative effects of glucocorticoids on muscle membrane stability have been reported [11]. This discrepancy may be due to the shorter treatment time (15 min) utilized for their assays, while in our study, a 20 hour treatment time was necessary to obtain the effect. Furthermore, glucocorticoids are known to regulate the synthesis and function of annexin A1 and exert an anti-inflammatory effect in non-muscle cells [52]. Annexin A1 is a member of a superfamily of annexin protein family that bind acidic phospholipids in the presence of Ca^2+^ and is known to be involved in a broad range of molecular processes [52]. Annexin A1 is activated by Ca^2+^ influx, binds to membrane phospholipids, and promotes membrane fusion and aggregation as well as the plasma membrane repair process [51]. For skeletal muscle, annexin A1 has been only reported to regulate myoblast migration and differentiation [64]. However, *annexin A6*, an *annexin A1*-related gene, has been recently identified as a modifier and a membrane repair factor of Limb-Girdle muscular dystrophy [65]. In our study, we clearly demonstrate that treatment with corticosterone can enhance the muscle membrane repair system in DMD model mice via up-regulation of annexin A1.

## Conclusions

Glucocorticoids have been widely utilized for therapeutic purposes, including those for DMD [11]. Our findings can give insight into the yet unclear effects of glucocorticoids in DMD. This may lead us towards a more beneficial avenue of DMD therapy, since glucocorticoids are the most established and the only medication available known to slow the decline in muscle strength and function in DMD patients.

## Supporting Information

S1 Fig List of primer pairs used for PCR experiments. Right columns denote predicted base pairs (bp) of DNA fragment sizes.
(TIF)

S2 Fig Histological characterizations of female *mdx* mice. (A) Area of fibrosis was analyzed by Sirius red staining for the virgin (n = 4) and PD2 (n = 4) *mdx* mice. (B) CD45-positive infiltrated cells per total diaphragm area was counted for the virgin (n = 4) and PD2 (n = 4) *mdx* mice. Bar (20 μm). (C) The number of slow muscle fibers stained by anti-slow MHC antibody was counted for the virgin (n = 4) and PD2 (n = 3) *mdx* mice. Bar (50 μm). (D) The number of CD31-positive vessels in diaphragm was counted for the virgin (n = 3) and PD2 (n = 3) *mdx* mice. Bar (200 μm). (E) Western blotting analysis detecting utrophin in the virgin (n = 3) and PD2 (n = 3) *mdx* mice diaphragm. Beta-tubulin was used for loading control. (F) Dystrophin-positive revertant fibers (white arrow) in the diaphragm was counted for the PD2 (n = 4) compared to virgin (n = 5) *mdx* mice. Bar (20 μm).
(TIF)

S3 Fig Number of muscle fibers used for hypo-osmotic shock experiments. (A) The values indicate total number of fibers (male and female wild-type and *mdx*) used for experiments, fibers survived after 5 or 20 hours pre-incubation (pre-shock) with vehicle, corticosterone, estradiol progesterone, and after 100% medium or 75% medium for hypo-osmotic shock (post-shock) following pre-incubation.
(TIF)

S4 Fig Screening of molecules with hypo-osmotic shock in isolated muscle fibers. (A) The relative survival rate of the fibers after 20 hours incubation with vehicle (n = 10), corticosterone (28.8 μM, n = 10), estradiol (27.5 μM, n = 5) or progesterone (27.6 μM, n = 5) before hypo-osmotic shock. (B) There was no increase in the relative survival rate of the fibers after 5 hours pre-incubation with corticosterone (28.8 μM, n = 4) after hypo-osmotic shock compared with vehicle (n = 4).
(TIF)
