# Supplementary figures and images for: Pregnancy-Induced Amelioration of Muscular Dystrophy Phenotype in mdx Mice via Muscle Membrane Stabilization Effect of Glucocorticoid
Source: PLoS One. 2015 Mar 16;10(3):e0120325. doi: 10.1371/journal.pone.0120325 (PMC4361742; doi:10.1371/journal.pone.0120325)

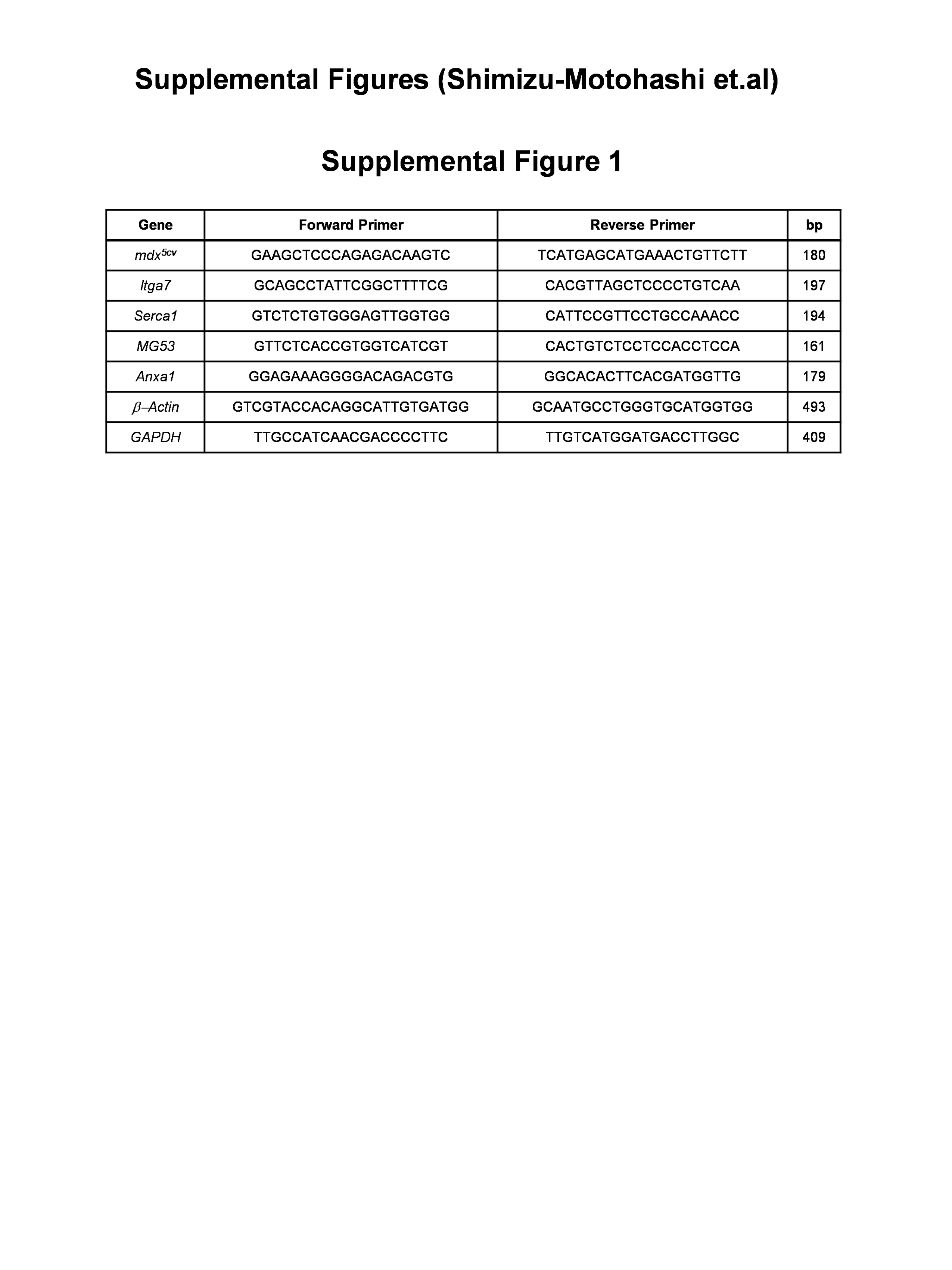

Supplement: S1 Fig — Right columns denote predicted base pairs (bp) of DNA fragment sizes. (TIF) [file pone.0120325.s001.tif]

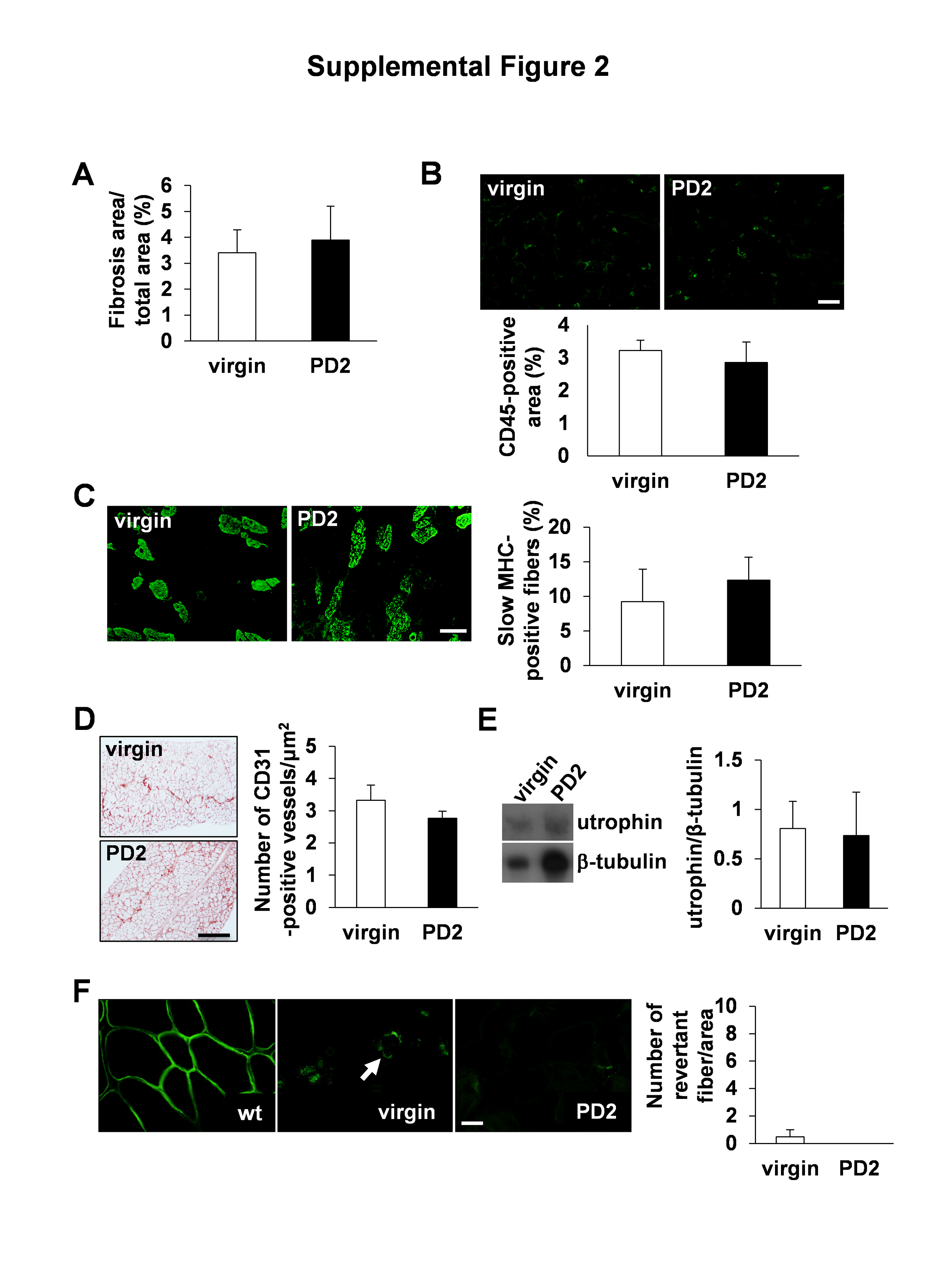

Supplement: S2 Fig — (A) Area of fibrosis was analyzed by Sirius red staining for the virgin (n = 4) and PD2 (n = 4) mdx mice. (B) CD45-positive infiltrated cells per total diaphragm area was counted for the virgin (n = 4) and PD2 (n = 4) mdx mice. Bar (20 μm). (C) The number of slow muscle fibers stained by anti-slow MHC antibody was counted for the virgin (n = 4) and PD2 (n = 3) mdx mice. Bar (50 μm). (D) The number of CD31-positive vessels in diaphragm was counted for the virgin (n = 3) and PD2 (n = 3) mdx mice. Bar (200 μm). (E) Western blotting analysis detecting utrophin in the virgin (n = 3) and PD2 (n = 3) mdx mice diaphragm. Beta-tubulin was used for loading control. (F) Dystrophin-positive revertant fibers (white arrow) in the diaphragm was counted for the PD2 (n = 4) compared to virgin (n = 5) mdx mice. Bar (20 μm). (TIF) [file pone.0120325.s002.tif]

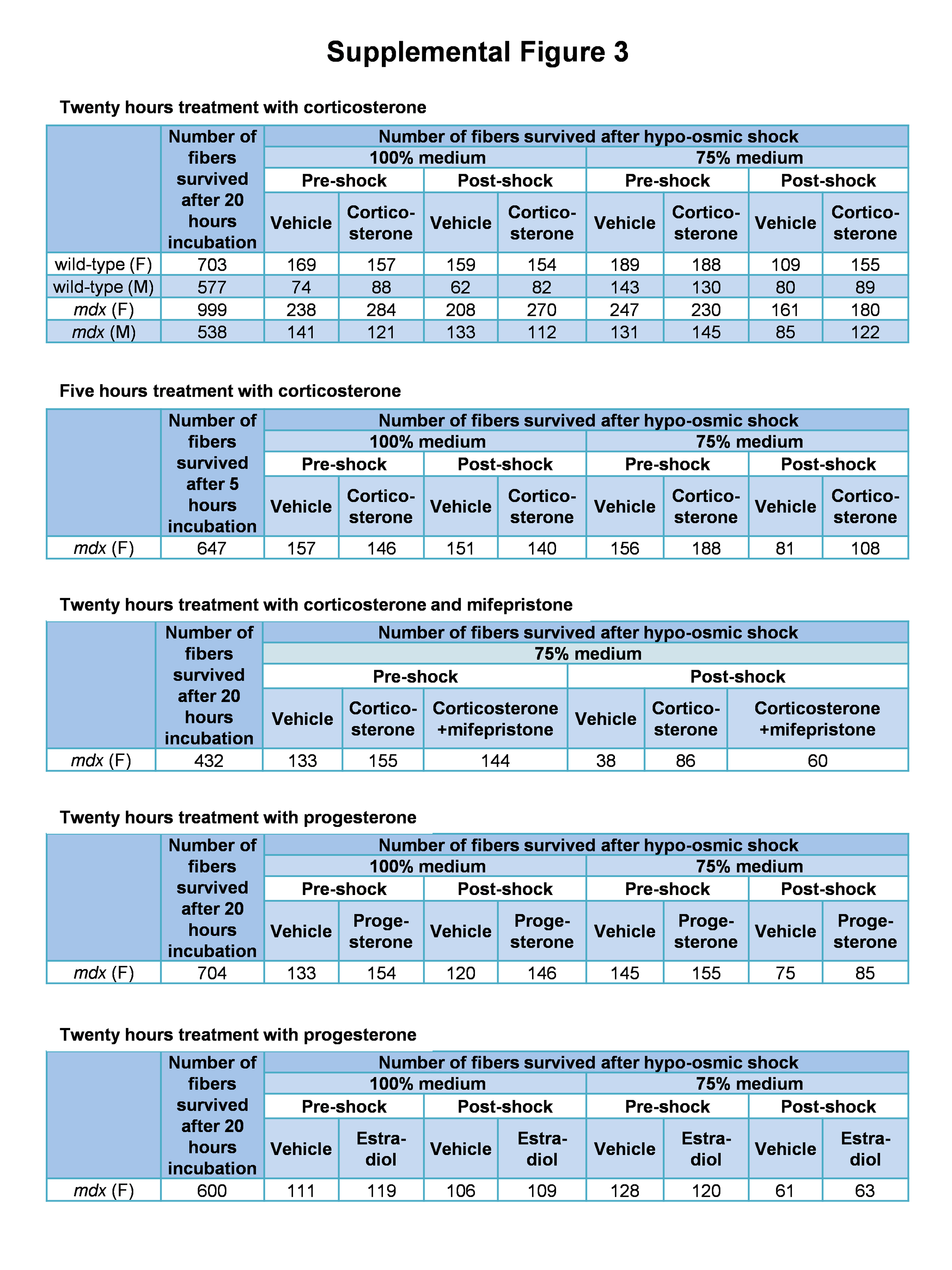

Supplement: S3 Fig — (A) The values indicate total number of fibers (male and female wild-type and mdx) used for experiments, fibers survived after 5 or 20 hours pre-incubation (pre-shock) with vehicle, corticosterone, estradiol progesterone, and after 100% medium or 75% medium for hypo-osmotic shock (post-shock) following pre-incubation. (TIF) [file pone.0120325.s003.tif]

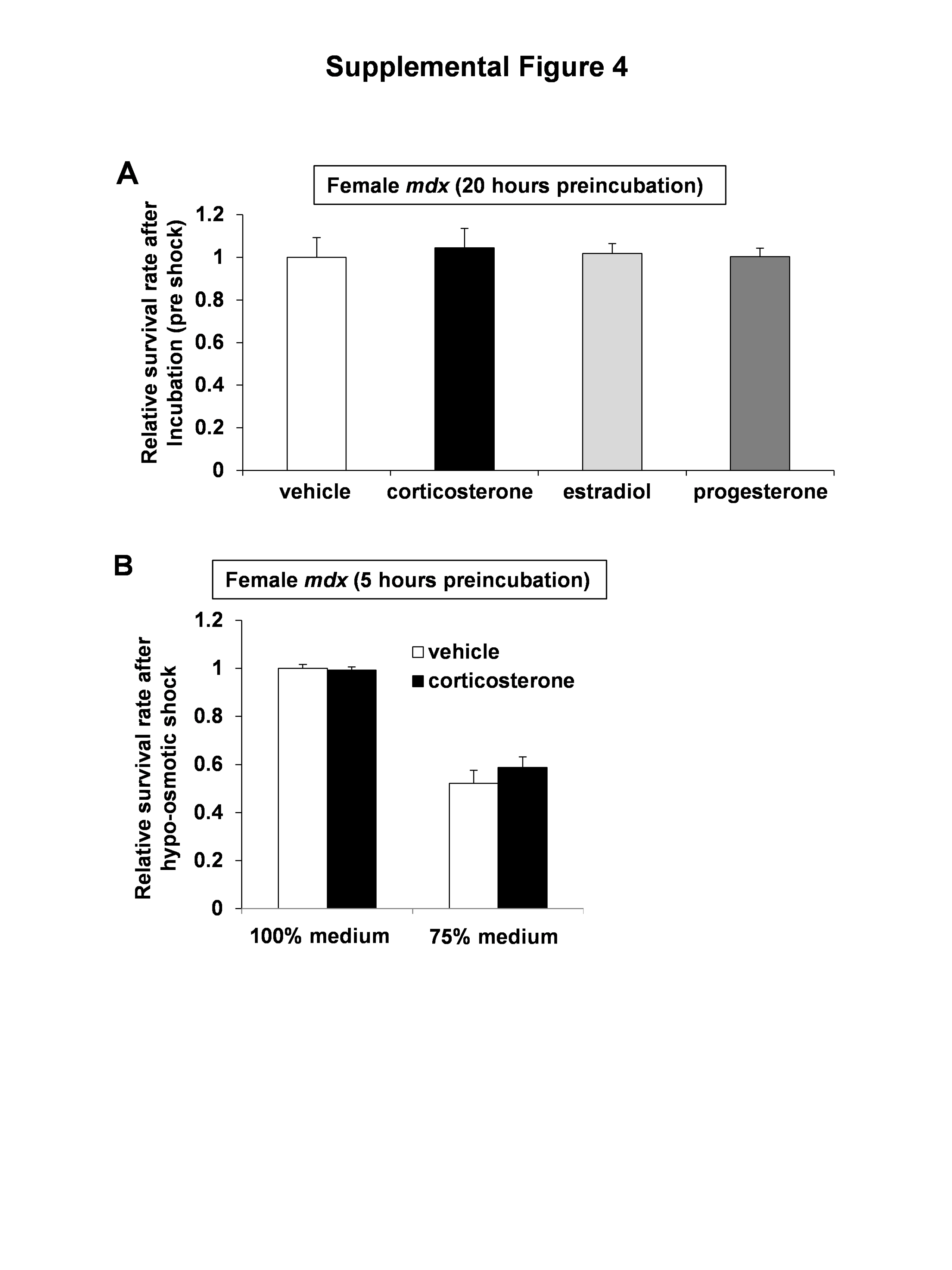

Supplement: S4 Fig — (A) The relative survival rate of the fibers after 20 hours incubation with vehicle (n = 10), corticosterone (28.8 μM, n = 10), estradiol (27.5 μM, n = 5) or progesterone (27.6 μM, n = 5) before hypo-osmotic shock. (B) There was no increase in the relative survival rate of the fibers after 5 hours pre-incubation with corticosterone (28.8 μM, n = 4) after hypo-osmotic shock compared with vehicle (n = 4). (TIF) [file pone.0120325.s004.tif]
